# Sixteenth Commencement of the Southern Medical College

**Published:** 1894-04

**Authors:** 


### SIXTEENTH COMMENCEMENT OF THE SOUTHERN MEDICAL COLLEGE.

The commencement exercises of the Southern Medical College were held at DeGive's Opera House on March 30th inst. This was the fifteenth anniversary of this institution, and the largest class that has ever graduated from this college. There were forty-two graduates. In presenting his report, Dr. Nicolson said:

*To the Board of Trustees, Southern Medical College:*

GENTLEMEN—At the conclusion of the fifteenth annual session of this institution we are much gratified to report that a very full measure of success has attended upon the efforts of the faculty, and that there have been many advances made in the direction of improvements in the course, as well as the equipment of the college.

At the last commencement we foreshadowed the increase in the length of course then contemplated, and now we have the pleasure to report that since that time, in common with the advanced schools of the country, we have increased the demands of attendance, as requisite to graduation, to three instead of two terms of lectures, and the length of each term has been increased to six instead of five months. We feel that the faculty deserve some credit for this step, as the most immediate competitor of the school in this city still requires a short course of only ten months, as opposed to our eighteen. It has been taken as a matter of conscience and as justly due to the people who thus receive from our hands better educated and more competent physicians.

It is especially gratifying that in the face of the increased requirements for graduation and the high standard required, as compared with other schools, the attendance for the session has been increased over the last term, and the class presented for graduation is the largest by far in the history of the school. This has demonstrated that the best students of this section are looking for higher education in medical branches, and also demonstrates the fact that what was considered at the time a very bold step was in the proper direction, and has been appreciated by the medical profession.

We present to you to-night as entitled to the degree of doctor of medicine forty-two young gentlemen, and can truly say, after rigid examination, that they fully deserve the degree.

Respectfully submitted,

WILLIAM PERRIN NICOLSON, Dean.

Dr. Thos. S. Powell, the venerable President and founder of the college awarded the diplomas to the graduates. His remarks were very dignified and impressive.

The valedictory address was a very fine one, and was delivered by Dr. W. J. Little, of Sparta, Ga.

Dr. R. S. Barrett, of St. Luke's Cathedral delivered the annual oration. It is useless to say that Dr. Barrett is one of the finest speakers in the South.

Dr. Nicolson, at the close of Dr. Barrett's address, announced the awards as follows:

First Honor—Dr. C. Pelham Ward.

Second Honor—Dr. Edward L. Brooks.

Third Honor—Dr. Isaac B. Diamond.

Professor Crichton's individual prize was awarded to Dr. Thomas J. McArthur.

Professor Roy's case of instruments was awarded to Dr. J. Brown Evans.

Drs. Evans, Harris, Lindsay, Little, O'Dell, Fritts and Huss were honorably mentioned.

The following are the members of the graduating class:

E. L. Brooks, T. W. Colvard, E. R. Corson, I. B. Diamond, A. B. Evans, Jr., J. H. Fritts, J. S. Fritts, E. L. Griffin, A. L. Harris, S. T. Harris, A. R. Holderby, B. F. Holliday, J. F. Huss, R. L. King, N. J. Langston, J. A Lindsay, W. J. Little, T. J. McArthur, G. S. Means, F. P. Nisbet, T. A. O'Dell, B. I. O'Kelly, G. M. Parker, W. H. Parker, H. W. Pharr, C. D. Redding, G. N. Ricketson, W. H. Rusk, W. B. Sharp, Henry B. Stanley, G. F. Taylor, J. L. Taylor, H. E. Thornton, A. H. Vandyke and C. P. Ward.

